# The genome sequence of the European crab apple, *Malus sylvestris* **(L.) Mill., 1768

**DOI:** 10.12688/wellcomeopenres.18645.1

**Published:** 2022-12-07

**Authors:** Markus Ruhsam, David Bell, Michelle Hart, Peter Hollingsworth, Royal Botanic Garden Edinburgh Genome Acquisition Lab, Darwin Tree of Life Barcoding collective, Wellcome Sanger Institute Tree of Life programme, Wellcome Sanger Institute Scientific Operations: DNA Pipelines collective, Tree of Life Core Informatics collective, Darwin Tree of Life Consortium

**Affiliations:** 1 Royal Botanic Garden, Edinburgh, Edinburgh, UK

**Keywords:** Malus sylvestris, European crab apple, genome sequence, chromosomal, Rosaceae

## Abstract

We present a genome assembly from an individual *Malus sylvestris* (the European or 'wild' crab apple; Streptophyta; Magnoliopsida; Rosales; Rosaceae). The genome sequence is 642 megabases in span. Most of the assembly (99.98%) is scaffolded into 17 chromosomal pseudomolecules. The mitochondrial and chloroplast genomes were also assembled, with respective lengths of 396.9 kilobases and 160.0 kilobases.

## Species taxonomy

Eukaryota; Viridiplantae; Streptophyta; Embryophyta; Tracheophyta; Spermatophyta; Magnoliopsida; eudicotyledons; Gunneridae; Pentapetalae; rosids; fabids; Rosales; Rosaceae; Amygdaloideae; Maleae; *Malus*; *Malus sylvestris* (L.) Mill., 1768 (NCBI:txid3752).

## Background

*Malus sylvestris* (European wild or crab apple) belongs to the Rose family (Rosaceae) and is a small tree native across Europe. It reaches its north-western distribution limit in Scotland and Norway and is probably the UK’s least understood and most under-appreciated tree, as even foresters sometimes do not seem to be aware of its native status. There is evidence of its longstanding use, as wild apples have been found at Neolithic and Bronze Age archaeological sites across Europe ( Cornille *et al.* 2014). Additionally, without the European wild apple, the eating apples so many people love would not be quite the same. It is one of the main contributors to the domesticated apple, *M. domestica* ( Cornille *et al.*, 2014; Cornille *et al.*, 2019). The widely planted cultivated varieties of *M. domestica* continue to hybridise with the wild apple trees, causing concern that the genetic integrity of *M. sylvestris* might be eroded in the long run ( Cornille *et al.*, 2013; Feurtey *et al.*, 2017; Ruhsam *et al.*, 2019; Schnitzler *et al.*, 2014).

In a recent study, nearly 30% of the wild apple trees genotyped turned out to be of hybrid origin ( Ruhsam *et al.*, 2019). As morphological identification of hybrid trees is difficult, access to the full genome of both *M. sylvestris* and *M. domestica* will facilitate the development of genome-wide species-specific markers, enabling the reliable assessment of levels of introgression in the European wild apple.

The genome of the European crab apple, *M. sylvestris,* was sequenced as part of the Darwin Tree of Life Project, a collaborative effort to sequence all named eukaryotic species in the Atlantic Archipelago of Britain and Ireland.

## Genome sequence report

**Figure 1.**
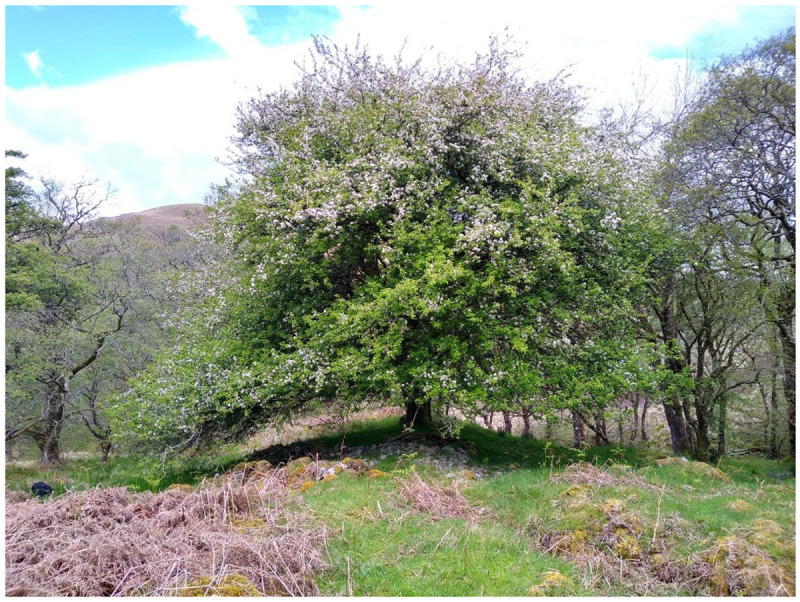
Image of the *Malus sylvestris* specimen from which samples used for sequencing were taken. The drMalSylv7 leaf samples were used to generate PacBio, 10X genomics and Hi-C data.

The genome was sequenced from a single *M. sylvestris* (hermaphroditic) ( Figure 1), collected from Glen Falloch, Scotland, UK. A total of 25-fold coverage in Pacific Biosciences single-molecule HiFi long reads and 87-fold coverage in 10X Genomics read clouds were generated. Primary assembly contigs were scaffolded with chromosome conformation Hi-C data. Manual assembly curation corrected 64 missing/misjoins and removed 10 haplotypic duplications, reducing the assembly size by 0.17% and the scaffold number by 36.0%.

**Figure 2.**
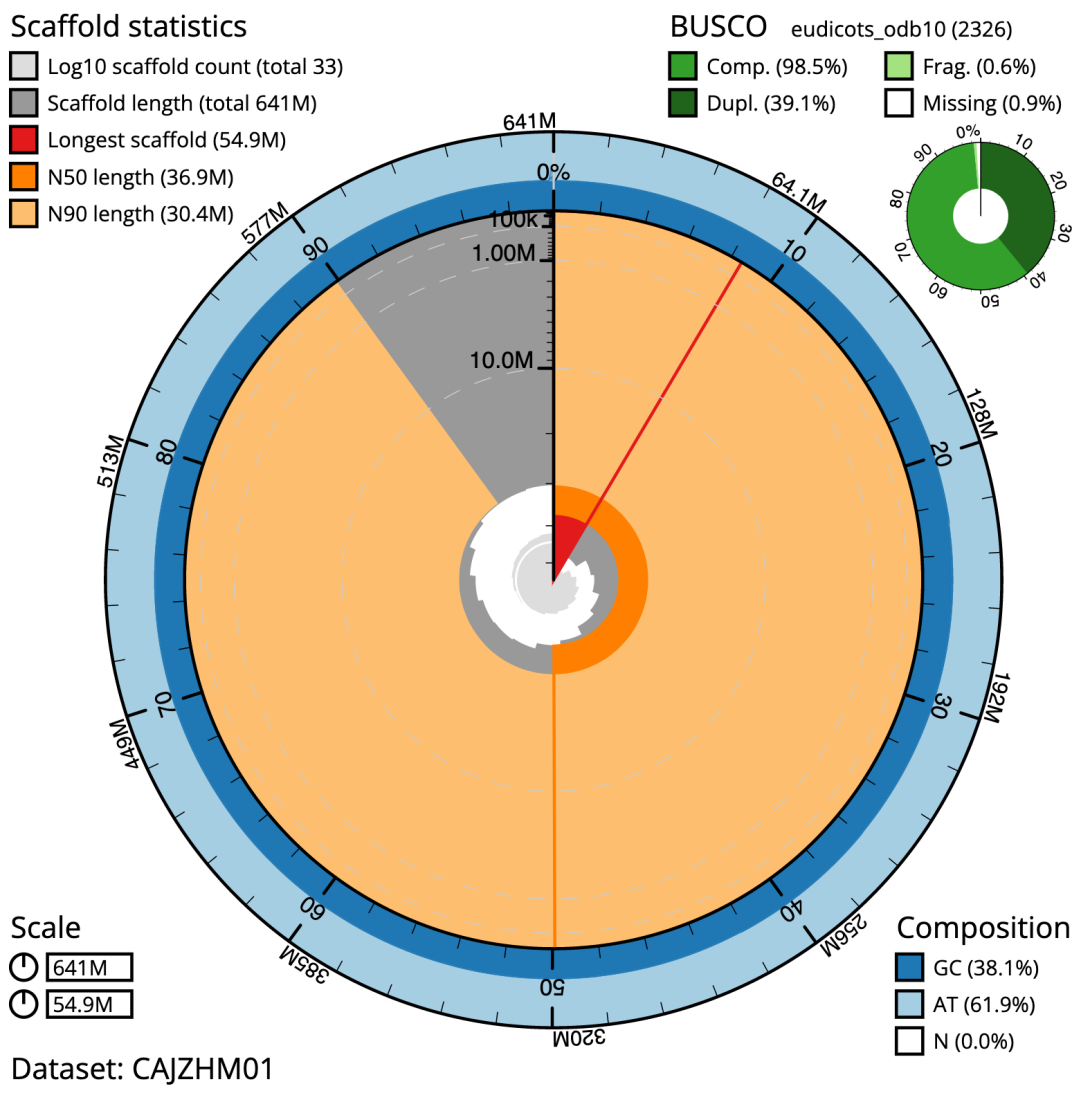
Genome assembly of *Malus sylvestris*, drMalSylv7.1: metrics. The BlobToolKit Snailplot shows N50 metrics and BUSCO gene completeness. The main plot is divided into 1,000 size-ordered bins around the circumference with each bin representing 0.1% of the 640,986,597 bp assembly. The distribution of chromosome lengths is shown in dark grey with the plot radius scaled to the longest chromosome present in the assembly (54,898,662 bp, shown in red). Orange and pale-orange arcs show the N50 and N90 chromosome lengths (36,902,754 and 30,380,474 bp), respectively. The pale grey spiral shows the cumulative chromosome count on a log scale with white scale lines showing successive orders of magnitude. The blue and pale-blue area around the outside of the plot shows the distribution of GC, AT and N percentages in the same bins as the inner plot. A summary of complete, fragmented, duplicated and missing BUSCO genes in the eudicots_odb10 set is shown in the top right. An interactive version of this figure is available at https://blobtoolkit.genomehubs.org/view/drMalSylv7.1/dataset/CAJZHM01/snail.

The final assembly has a total length of 641 Mb in 32 sequence scaffolds with a scaffold N50 of 36.9 Mb ( Table 1). Most of the assembled sequence (99.98%) was assigned to 17 chromosomal-level scaffolds numbered by synteny based on *Malus domestica* (apple) GCA_004115385.1 ( Figure 2– Figure 5; Table 2).

**Table 1.** Genome data for *Malus sylvestris*, drMalSylv7.2.

*Project accession data*	
Assembly identifier	drMalSylv7.2
Species	*Malus sylvestris*
Specimen	drMalSylv7 (genome assembly, Hi-C)
NCBI taxonomy ID	3752
BioProject	PRJEB47316
BioSample ID	SAMEA9197672
Isolate information	Leaf tissue
*Raw data accessions*	
PacificBiosciences SEQUEL II	ERR6808038, ERR6808039
10X Genomics Illumina	ERR6688721-ERR6688723; ERR6688403
Hi-C Illumina	ERR6688724
Standard Illumina libraries	ERR6909395-ERR6909404
*Genome assembly*	
Assembly accession	GCA_916048215.2
*Accession of alternate haplotype*	GCA_916049865.1
Span (Mb)	641
Number of contigs	133
Contig N50 length (Mb)	8.3
Number of scaffolds	32
Scaffold N50 length (Mb)	36.9
Longest scaffold (Mb)	54.9
BUSCO * genome score	C:98.5%[S:59.4%,D:39.1%], F:0.6%,M:0.9%,n:2,326

*BUSCO scores based on the eudicots_odb10 BUSCO set using v5.3.2. C = complete [S = single copy, D = duplicated], F = fragmented, M = missing, n = number of orthologues in comparison. A full set of BUSCO scores is available at https://blobtoolkit.genomehubs.org/view/drMalSylv7.1/dataset/CAJZHM01/busco.

**Figure 3.**
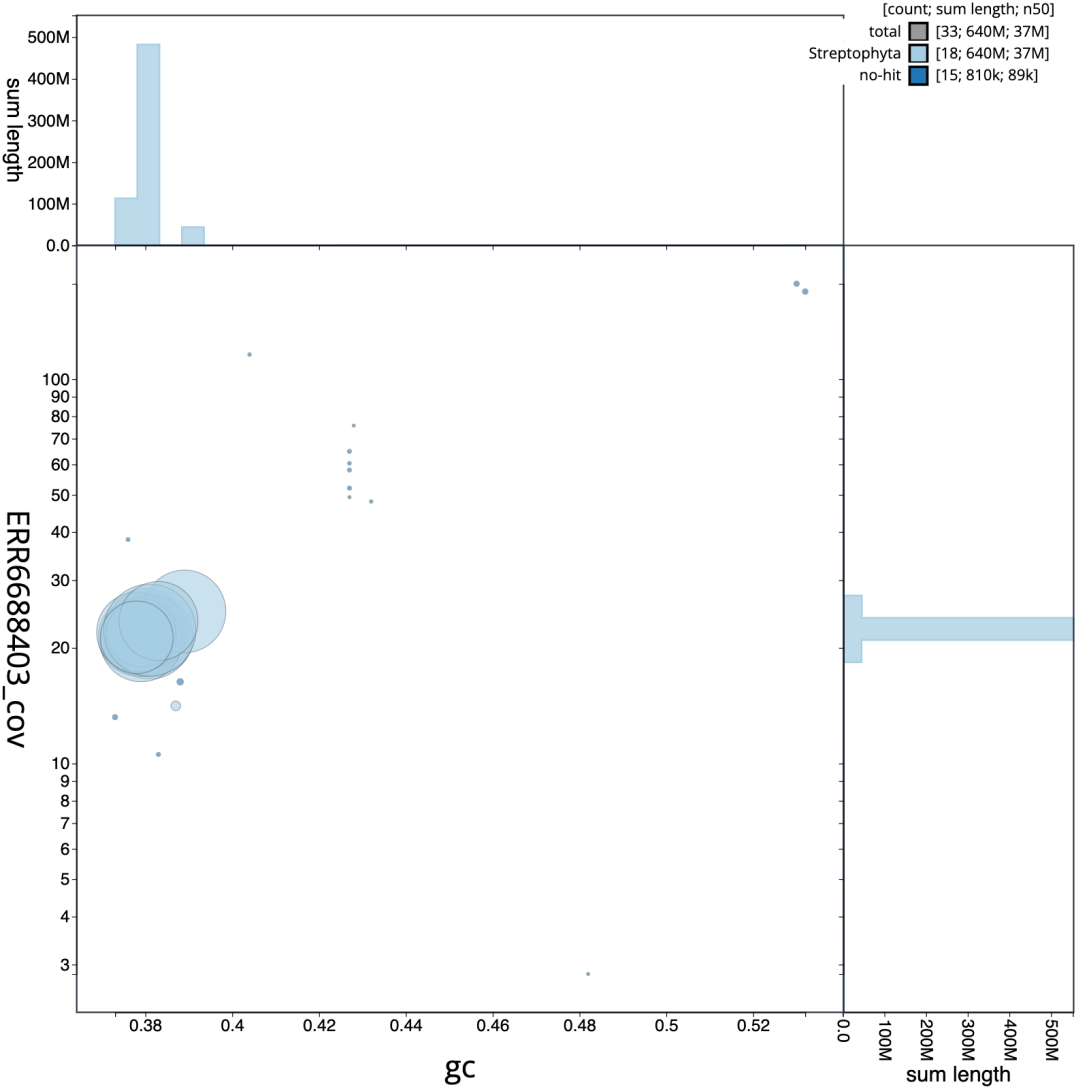
Genome assembly of *Malus sylvestris*, drMalSylv7.1: GC coverage. BlobToolKit GC-coverage plot. Scaffolds are coloured by phylum. Circles are sized in proportion to scaffold length. Histograms show the distribution of scaffold length sum along each axis. An interactive version of this figure is available at https://blobtoolkit.genomehubs.org/view/drMalSylv7.1/dataset/CAJZHM01/blob.

**Figure 4.**
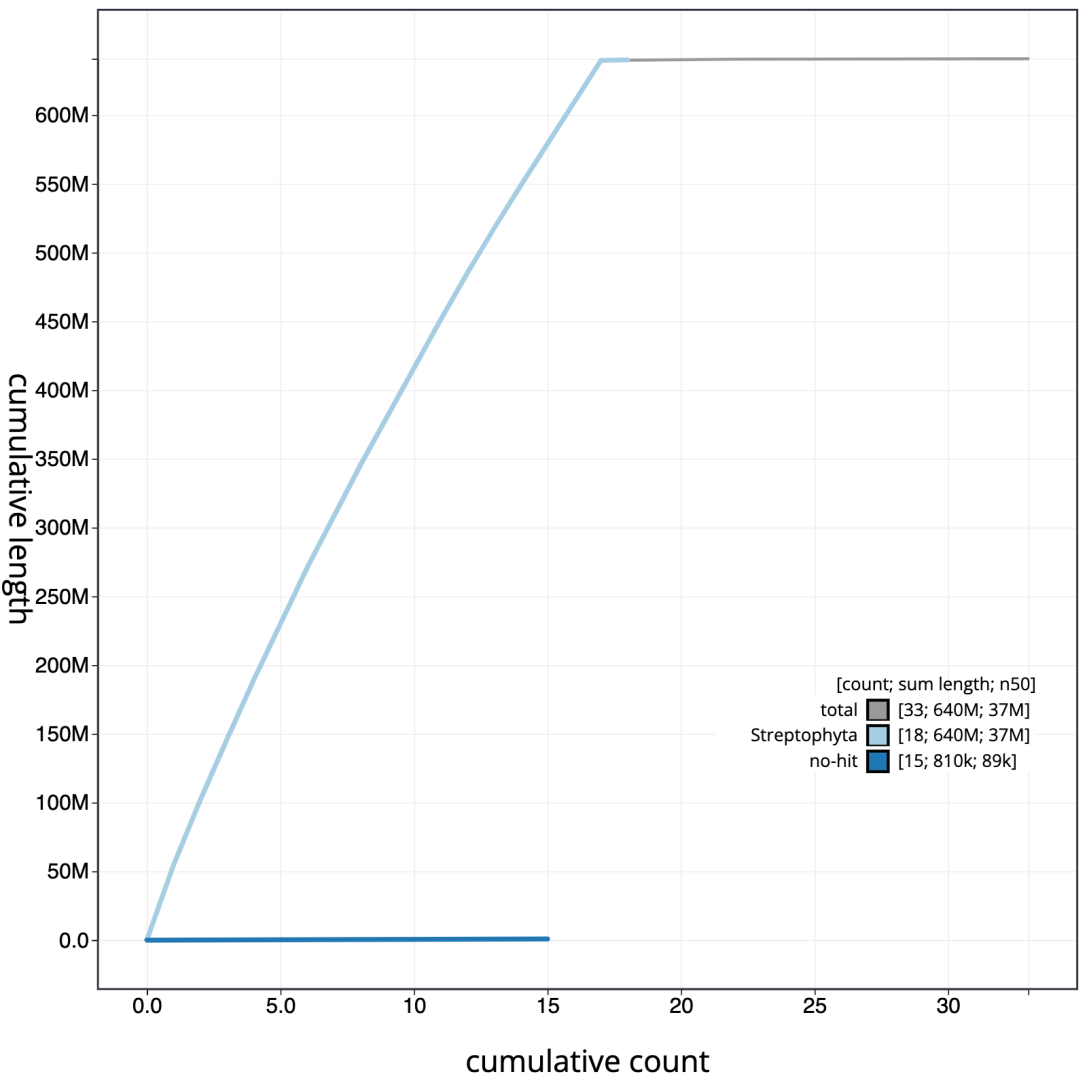
Genome assembly of *Malus sylvestris*, drMalSylv7.1: cumulative sequence. BlobToolKit cumulative sequence plot. The grey line shows cumulative length for all scaffolds. Coloured lines show cumulative lengths of scaffolds assigned to each phylum using the buscogenes taxrule. An interactive version of this figure is available at https://blobtoolkit.genomehubs.org/view/drMalSylv7.1/dataset/CAJZHM01/cumulative.

**Figure 5.**
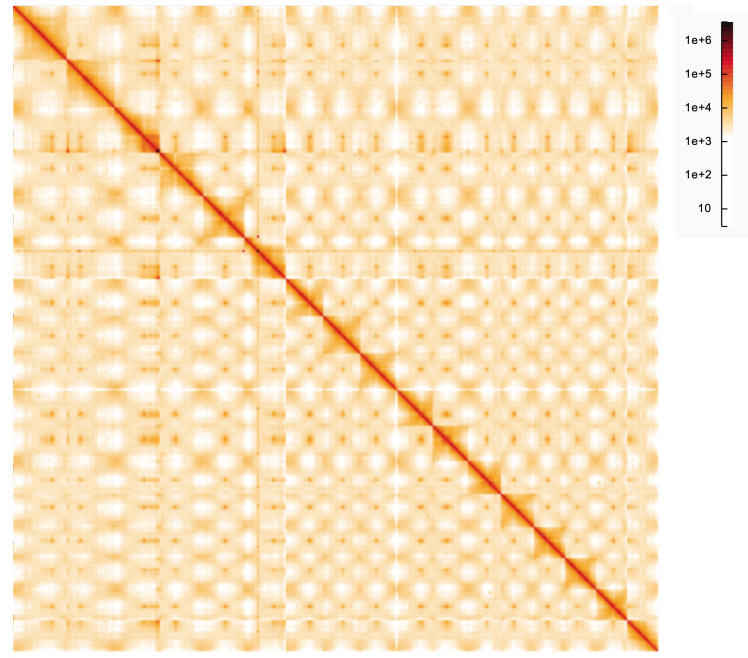
Genome assembly of *Malus sylvestris*, drMalSylv7.2: Hi-C contact map. Hi-C contact map of the drMalSylv7.2 assembly, visualised in HiGlass. Chromosomes are arranged in size order from left to right and top to bottom. The interactive Hi-C map can be viewed at https://genome-note-higlass.tol.sanger.ac.uk/l/?d=FgGkT9v7SR2otUnBK-HkfQ.

**Table 2.** Chromosomal pseudomolecules in the genome assembly of *Malus sylvestris*, drMalSylv7.2. Chromosome-scale scaffolds are numbered by synteny based on *Malus domestica* (apple) GCA_004115385.1.

INSDC accession	Name	Size (Mb)	GC%
OU696503.1	1	30.13	38.3
OU696504.1	2	37.96	37.8
OU696505.1	3	36.9	38
OU696506.1	4	31.14	38
OU696507.1	5	47.13	38.1
OU696508.1	6	34.96	38
OU696509.1	7	35.53	37.9
OU696510.1	8	30.37	37.9
OU696511.1	9	35.18	38.1
OU696512.1	10	43.2	37.9
OU696513.1	11	41.35	37.8
OU696514.1	12	32.55	37.9
OU696515.1	13	44.07	38.9
OU696516.1	14	30.38	37.9
OU696517.1	15	54.9	38.1
OU696518.1	16	40.12	38.3
OU696519.1	17	33.87	37.8
-	MT	0.4	45.4
-	Pltd	0.16	36.6
-	unplaced	1.23	41.7

Shared sequences between chromosomes are visible in the Hi-C map in agreement with the findings of ( Velasco *et al.*, 2010) for *Malus domestica*. The Hi-C map provides evidence of inversions between haplotypes in chromosome 2 (24.82–26.19 Mb), and chromosome 11 (17.06–19.05 Mb). There are several scaffolds that it was possible to localise to a chromosome but not place, and these have been labelled as ‘unloc’. Two of the largest unlocalised scaffolds – SUPER_4_unloc_1 and SUPER_14_unloc_1 – appear to be larger haplotypes of two specific loci: Chromosome 4 at ~26.13–26.31 Mb and chromosome 14 at ~782 kb respectively. From the Hi-C it appears that these loci currently represent the shorter haplotype. As there is some uncertainty over these unlocalised scaffolds, they were left in the primary assembly.

The assembly has a BUSCO v5.3.2 ( Manni *et al.*, 2021) completeness of 98.5% (single 59.4%, duplicated 39.1%) using the eudicots_odb10 reference set ( *n* = 2,326). While not fully phased, the assembly deposited is of one haplotype. Contigs corresponding to the second haplotype have also been deposited.

## Methods

### Sample acquisition and nucleic acid extraction

Leaf samples from a single *M. sylvestris* specimen (drMalSylv7; genome assembly, Hi-C) were collected from Glen Falloch, Scotland, UK (latitude 56.343794, longitude –4.7078054) by Markus Ruhsam (Royal Botanic Garden Edinburgh), who also identified the specimen. The leaf samples were picked by hand, cut and put into FluidX tubes, then snap-frozen in liquid nitrogen.

The drMalSylv7 leaf sample was weighed and dissected on dry ice with tissue set aside for Hi-C sequencing. Leaf tissue was cryogenically disrupted to a fine powder using a Covaris cryoPREP Automated Dry Pulveriser, receiving multiple impacts. High molecular weight (HMW) DNA was extracted using the Qiagen Plant MagAttract HMW DNA extraction kit. Low molecular weight DNA was removed from a 200 ng aliquot of extracted DNA using 0.8X AMpure XP purification kit prior to 10X Chromium sequencing; a minimum of 50 ng DNA was submitted for 10X sequencing. HMW DNA was sheared into an average fragment size of 12–20 kb in a Megaruptor 3 system with speed setting 30. Sheared DNA was purified by solid-phase reversible immobilisation using AMPure PB beads with a 1.8X ratio of beads to sample to remove the shorter fragments and concentrate the DNA sample. The concentration of the sheared and purified DNA was assessed using a Nanodrop spectrophotometer and Qubit Fluorometer and Qubit dsDNA High Sensitivity Assay kit. Fragment size distribution was evaluated by running the sample on the FemtoPulse system.

### Sequencing

Pacific Biosciences HiFi circular consensus and 10X Genomics Chromium read cloud sequencing libraries were constructed according to the manufacturers’ instructions. Sequencing was performed by the Scientific Operations core at the Wellcome Sanger Institute on Pacific Biosciences SEQUEL II (HiFi) and Illumina NovaSeq 6000 (10X) instruments. Hi-C data were generated in the Tree of Life laboratory from remaining leaf tissue of drMalSylv7 using the Arima v2 kit and sequenced on a NovaSeq 6000 instrument. Standard read sequencing libraries were generated using an Illumina NovaSeq 6000 (10X) instrument, as per the manufacturer’s instructions.

### Genome assembly

Assembly was carried out with Hifiasm ( Cheng *et al.*, 2021) and haplotypic duplication was identified and removed with purge_dups ( Guan *et al.*, 2020). One round of polishing was performed by aligning 10X Genomics read data to the assembly with longranger align, calling variants with freebayes ( Garrison & Marth, 2012). The assembly was then scaffolded with Hi-C data ( Rao *et al.*, 2014) using SALSA2 ( Ghurye *et al.*, 2019). The assembly was checked for contamination as described previously ( Howe *et al.*, 2021). Manual curation was performed using HiGlass ( Kerpedjiev *et al.*, 2018) and Pretext ( Harry, 2022). The mitochondrial and plastid genomes were assembled using MBG ( Rautiainen & Marschall, 2021) from PacBio HiFi reads mapping to related genomes. A representative circular sequence was selected for each from the graph based on read coverage. The genome was analysed and BUSCO scores were generated within the BlobToolKit environment ( Challis *et al.*, 2020). Table 3 contains a list of the software tool versions used, where appropriate.

**Table 3.** Software tools used.

Software tool	Version	Source
Hifiasm	0.15.3-r339	Cheng *et al.*, 2021
purge_dups	1.2.3	Guan *et al.*, 2020
SALSA2	2.2	Ghurye *et al.*, 2019
longranger align	2.2.2	https://support.10xgenomics.com/ genome-exome/software/pipelines/ latest/advanced/other-pipelines
freebayes	1.3.1-17- gaa2ace8	Garrison & Marth, 2012
HiGlass	1.11.6	Kerpedjiev *et al.*, 2018
PretextView	0.2.x	Harry, 2022
BlobToolKit	3.2.9	Challis *et al.*, 2020

### Ethics/compliance issues

The materials that have contributed to this genome note have been supplied by a Darwin Tree of Life Partner. The submission of materials by a Darwin Tree of Life Partner is subject to the Darwin Tree of Life Project Sampling Code of Practice. By agreeing with and signing up to the Sampling Code of Practice, the Darwin Tree of Life Partner agrees they will meet the legal and ethical requirements and standards set out within this document in respect of all samples acquired for, and supplied to, the Darwin Tree of Life Project. Each transfer of samples is further undertaken according to a Research Collaboration Agreement or Material Transfer Agreement entered into by the Darwin Tree of Life Partner, Genome Research Limited (operating as the Wellcome Sanger Institute), and in some circumstances other Darwin Tree of Life collaborators.

## Data Availability

European Nucleotide Archive: *Malus sylvestris* (European crab apple). Accession number PRJEB47316; https://identifiers.org/ena.embl/PRJEB47316 ( Wellcome Sanger Institute, 2022) The genome sequence is released openly for reuse. The *M. sylvestris* genome sequencing initiative is part of the Darwin Tree of Life (DToL) project. All raw sequence data and the assembly have been deposited in INSDC databases. The genome will be annotated and presented through the Ensembl pipeline at the European Bioinformatics Institute. Raw data and assembly accession identifiers are reported in Table 1.

## Author information

Members of the Royal Botanic Garden Edinburgh Genome Acquisition Lab are listed here: https://doi.org/10.5281/zenodo.4786682.

Members of the Darwin Tree of Life Barcoding collective are listed here:

https://doi.org/10.5281/zenodo.4893703.

Members of the Wellcome Sanger Institute Tree of Life programme are listed here:

https://doi.org/10.5281/zenodo.4783585.

Members of Wellcome Sanger Institute Scientific Operations: DNA Pipelines collective are listed here: https://doi.org/10.5281/zenodo.4790455.

Members of the Tree of Life Core Informatics collective are listed here:

https://doi.org/10.5281/zenodo.5013541.

Members of the Darwin Tree of Life Consortium are listed here:

https://doi.org/10.5281/zenodo.4783558.
