# Supplementary material for: The genome sequence of the European crab apple, Malus sylvestris  (L.) Mill., 1768
Source: Wellcome Open Res. 2022 Dec 7;7:296. [Version 1] doi: 10.12688/wellcomeopenres.18645.1 (PMC9975420; doi:10.12688/wellcomeopenres.18645.1)
Supplement: Peer review history

# Peer review: Reviewer response for version 1

**DOI:** 10.21956/wellcomeopenres.20675.r54253

**Published:** 2023-01-25

**Authors:** Xilong Chen

**Affiliations:** 1 Génétique Quantitative et Évolution - Le Moulon , Gif sur Yvette, France

This article presents the assembly of the genome of the European wild apple ( *Malus sylvestris)* . The authors combined data from Pacific Biosciences, Illumina HiSeq 10X, and Hi-C to assemble the genome. The result has good assembly metrics N50 and BUSCO. The graphical information and the software listings help to understand the data and the reproducibility studies. The authors also compared the assembly results with the cultivated apple ( *Malus domestica* ) genome. Some structural variants were found.

 To conclude, this article is an important contribution to European wild apple genome research and the genomic data provide a basis for studying apple domestication and Rosaceae evolution. In addition, I hope that the authors will complete the annotation of the genes and make it publicly available.

Are sufficient details of methods and materials provided to allow replication by others?

Yes

Is the rationale for creating the dataset(s) clearly described?

Yes

Are the datasets clearly presented in a useable and accessible format?

Yes

Are the protocols appropriate and is the work technically sound?

Yes

Reviewer Expertise:

Population genetics, fruit tree biology, next generation sequencing

I confirm that I have read this submission and believe that I have an appropriate level of expertise to confirm that it is of an acceptable scientific standard.

# Peer review: Reviewer response for version 1

**DOI:** 10.21956/wellcomeopenres.20675.r53634

**Published:** 2023-01-06

**Authors:** Nimisha Sharma

**Affiliations:** 1 Division of Fruits and Horticultural Technology, ICAR-Indian Agricultural Research Institute, New Delhi, India

Assessing the genome of crab apple is very much useful for the apple breeders, as *M. domestica* is cultivated apple and wild apple is one of the contributors, to know the molecular mechanism behind any trait specific breeding and tailoring of the crop accordingly would be easier and their genetic mechanism can be interpreted.

- I feel abbreviations must be provided so that it will be more understandable to every discipline person.
- Reference genome assembly could be generated.
- Many times wild species are not taken into consideration but they may be very informative for cultivated apples and if breeders want to introgress any gene from the wild species, it could be possible with this genome.
- Present manuscript related with the sequence of apple genome and it is just 10X which is very low coverage.
- Figure 1 must focus on the studied species rather than background.
- Is this genome mapped with other published genomes of the Rosaceae family?

However, after revision present manuscript can be accepted as it imparts knowledge for other species as well.

Are sufficient details of methods and materials provided to allow replication by others?

Yes

Is the rationale for creating the dataset(s) clearly described?

Yes

Are the datasets clearly presented in a useable and accessible format?

Yes

Are the protocols appropriate and is the work technically sound?

Yes

Reviewer Expertise:

Molecular Biology of Fruit Crops

I confirm that I have read this submission and believe that I have an appropriate level of expertise to confirm that it is of an acceptable scientific standard.
